# Improving Rabbit Doe Metabolism and Whole Reproductive Cycle Outcomes via Fatty Acid-Rich *Moringa oleifera* Leaf Extract Supplementation in Free and Nano-Encapsulated Forms

**DOI:** 10.3390/ani12060764

**Published:** 2022-03-18

**Authors:** Nagwa I. El-Desoky, Nesrein M. Hashem, Ahmed G. Elkomy, Zahraa R. Abo-Elezz

**Affiliations:** Department of Animal and Fish Production, Faculty of Agriculture (El-Shatby), Alexandria University, Alexandria 21545, Egypt; enagwa278@gmail.com (N.I.E.-D.); ahmed.elkoumi@alexu.edu.eg (A.G.E.); mkosba@hotmail.com (Z.R.A.-E.)

**Keywords:** Moringa, encapsulation, reproduction, milk, fatty acid, immunoglobulins

## Abstract

**Simple Summary:**

Under intensive rabbit production systems, due to the increased energy requirements of reproductive events, specifically pregnancy and lactation, rabbit does may confront several metabolic disorders as a result of energy imbalance. *Moringa oleifera* leaf ethanolic extract (ME) is one of the phytogenic extracts that has an impressive range of phytochemicals, specifically fatty acids (FAs). These phytochemicals may be biologically effective to support metabolism and reproductive functions of rabbit does during different reproductive cycle events. However, the high FAs content of ME makes them highly susceptible to lipid oxidation, diminishing their nutritional value and biological effects. In this study, we aimed to test the effects of FAs of ME either in a free-from or in a nano-encapsulated form on metabolism, immunity, milk production, milk composition, and reproductive performance of rabbit does during different physiological status (premating, mating, pregnancy, and lactation). The results showed that ME improved health, metabolism, immune functions, milk production and composition, and reproductive performance of rabbit does. These effects remained obvious even when a lower dose of ME was used in a nano-encapsulated form.

**Abstract:**

The effects of free and nano-encapsulated ME supplementations on the metabolism, immunity, milk production and composition, and reproductive performance of rabbit does during premating, mating, pregnancy, and lactation were investigated. Multiparous rabbit does (*n* = 26 per group) received 50 mg of free ME (FME) daily, 25 mg of nano-encapsulated ME (HNME), or 10 mg of nano-encapsulated ME (LNME) per kilogram of body weight or were not supplemented (C) during a whole reproductive cycle. The ME contained 30 fatty acids with 54.27% total unsaturated fatty acids (USFAs). The fatty acid encapsulation efficiency of alginate nanoparticles was 70.46%. Compared with the C group, rabbits in all ME treatments had significantly increased body weight, feed intake, and glucose concentration and significantly decreased non-esterified free fatty acids and β-hydroxybutyrate concentrations. Rabbits supplemented with ME also had significantly increased white blood cell counts, phagocytic activity, lysozyme activity, and immunoglobulin G and decreased interleukin-1β concentrations. Moreover, ME supplementation significantly increased the concentrations of colostrum immunoglobulins, milk yield and energy content, and milk USFAs (omega-3 and 6). Rabbit does in the ME treatments had significantly higher conception and parturition rates and better litter characteristics than the C rabbit does. These results demonstrate the positive role of ME fatty acids on the health status and productive and reproductive performance of rabbit does at different physiological stages. Compared with the FME treatment, these parameters were further improved in rabbits that received nano-encapsulated ME at lower doses, illustrating how nano-encapsulation technology improves the bioavailability of ME.

## 1. Introduction

On rabbit farms, rabbit does are considered biological units used for producing new individuals; thus, their reproductive efficiency governs production and subsequently, economic returns. Rabbits are typically bred in intensive production systems, which results in the simultaneous occurrence of more than one reproductive event, mainly pregnancy and lactation. This intensive production system represents a great metabolic burden on rabbit does, as they must balance their nutritional requirements with pregnancy and lactation requirements to sustain milk production and/or the growth of the fetuses [1,2].

Imbalances in the nutritional/energy requirements of rabbit does can lead to serious metabolic disorders and negative energy balance, which decreases the reproductive performance of rabbit does [3]. The body condition and energy balance of rabbit does have been shown to affect their short-term and long-term reproductive efficiency [4]. If rabbit does lack adequate energy sources and nutritional requirements during their reproductive cycle, they may develop metabolic disorders, such as glycemia (shortage of circulating glucose levels), and exhibit increased levels of undesirable metabolites, such as non-esterified free fatty acids (NEFAs) and β-hydroxybutyrate (β-HB), due to body fat lipolysis [5,6]. Additionally, negative energy balances are associated with disrupted immunity and elevated inflammatory reactions [7].

Many attempts have been made to improve the body condition and energy status of rabbit does during sensitive reproductive windows using energy-rich diets. However, feed intake does not meet the energy deficit, leading to the mobilization of body reserves, lost body energy, and inadequate reproductive performance [5,8]. Functional nutrients/molecules, such as fatty acids (FAs), amino acids, and vitamins that can modulate metabolism and/or support physiological functions may represent an effective intervention [9,10]. Among these functional nutrients/molecules, FAs have many vital biological roles in mammals. The hepatocytes use FAs as precursors for synthesizing essential energy-yielding metabolites, such as triglycerides [11], and are used as an energy substrate by oocytes and embryos [12]. Moreover, FAs are necessary for gamete/embryo differentiation, growth, and immune system function [12] via several modes of action. Many FAs act as functional molecules for many reproductive events; numerous studies have reported the positive effects of FAs on ovulation, fertilization, pregnancy outcomes, and milk production in rabbits [9,13,14,15]. For example, the ovulation rate and the number of normal embryos are positively correlated with monounsaturated FAs (MUFAs) concentrations at the time of mating [12,15]. In the pre- and post-implantation periods of rabbit does, supplementation with docosahexaenoic acid methyl ester (DHA) and eicosapentaenoic acid methyl ester (EPA) increases plasma progesterone levels and improves pregnancy outcomes [16,17].

*Moringa oleifera* is a tropical plant rich in biologically active substances, including FAs, amino acids, vitamins, minerals, and phytochemicals. Several studies confirmed the enrichment of this plant with biologically active FAs, specifically polyunsaturated FAs (PUFAs) [18,19,20]. However, PUFAs are highly susceptible to lipid oxidation, which diminishes their nutritional value and biological effects. To overcome this problem, encapsulation processes can be used to protect products rich in PUFAs from the oxidation reactions that occur when PUFAs are exposed to oxygen, metal ions, high temperatures, and light [21].

In this study, the effects of FAs in either free-from or nano-encapsulated form of ME on metabolism, immunity, milk production, milk composition, and reproductive performance in rabbit does during different physiological stages (premating, mating, pregnancy, and lactation) were investigated.

## 2. Materials and Methods

This study was conducted at the Laboratory of Rabbit Physiology Research, Agricultural Experimental Station, Faculty of Agriculture, Alexandria University, Egypt. The experimental procedures were revised and approved by the Alexandria University-Institutional Animal Care and Use Committee (AU-IACUC) with approval number AU 08 21 07 26 2 82.

### 2.1. Moringa oleifera Leaf Extraction and Nanofabrication

Moringa (*Moringa oleifera*) leaves were extracted using 70% ethanol solution, filtrated, and evaporated to complete dryness. The dried ME was used to fabricate a sodium alginate nano-complex using calcium chloride (CaCl_2_) as a cross-linking agent by adopting the ionic-gelation method [22].

### 2.2. Nano-Encapsulated ME Physicochemical Properties and Fatty Acid Profile

In our previous study [22], we determined the physicochemical properties of alginate–CaCl_2_ nano-encapsulated ME assayed using a dynamic light scattering nanoparticle analyzer. The mean size of the fabricated nanoparticles was 93.69 nm, the zeta potential was 8.95 mV, and the polydispersity (PdI) was 0.442. The encapsulation efficiency of alginate–CaCl_2_ nanoparticles for ME phenolic compounds was 57.43% [22].

In this study, further analyses were conducted to identify the FA profile of ME and the FA encapsulation efficiency (EE) of the alginate–CaCl_2_ nanoparticles. For this purpose, 10 mL of each ME raw and supernatant (free ME, nonencapsulated fractions) sample was mixed with 50 mL of a methanol-chloroform mixture (2:1) in a separation funnel. The mixture was vigorously shaken for 5 min and left for separation. The mixture was centrifuged for 10 min at 2500 rpm to achieve layer separation. The chloroform layer was removed, filtered through a coarse filter paper, and evaporated until completely dry [23]. For fat methylation, a weight of 50 mg of lipids of both raw extracted ME and supernatant (free ME, nonencapsulated fractions obtained by centrifugation of the ME after the nano-encapsulation process) was incubated with 10 mL of a sulfuric acid (1%) and methanol mixture (1:100) in a water bath at 90 °C for 90 min. After cooling, 8 mL of water and 5 mL of petroleum ether were added, and the mixture was shaken vigorously. The ether layer was withdrawn and evaporated to dryness. FA methyl esters were quantified using a Thermo Scientific gas chromatograph GC Trace 1300 coupled with an EI Mass spectrometer ISQ 7000 model (Thermo Fisher Scientific, 168 Third Avenue Waltham, MA, USA) equipped with Thermo TR-50 MS capillary column (30 m in length × 250 μm in diameter × 0.25 μm in thickness of film). Spectroscopic detection by GC–MS involved an electron ionization system that utilized high energy electrons (70 eV), MS transfer line temperature 300 °C and ion source temperature 300 °C. Pure helium gas (99.995%) was used as the carrier gas with a flow rate of 1 mL/min. The initial temperature was set at 60 °C for 2 min, then increased to 100 °C at a rate of 10 °C/min kept for 5 min, then with 10 °C/min to 150 °C and kept for 5 min, then with 10 °C/min to 200 °C and kept for 5 min, then with 10 °C/min to 250 °C and kept for 20 min. One microliter of the prepared extracts was injected in a splitless mode. The concentrations of individual FAs in raw and supernatant of ME were calculated by dividing the relative area for each detected individual FA by the total area for Fas and expressed as g/100 g FA methyl esters [24]. The EE (%) of ME Fas by sodium alginate–CaCl_2_ nanoparticles were estimated by determining the FA concentration of each FA in the raw ME (before encapsulation, C raw) and the resultant supernatant following the collection of the nano-complex particles (C supernatant), using the following equation: EE (%) = C raw–C supernatant/C raw × 100.

### 2.3. Animal Management and Experimental Design

One hundred and four, multiparous, V-line rabbit does (a maternal synthetic line selected based on litter size at weaning [25]), weighing 2.75 ± 0.18 kg, were managed under similar housing (The average of ambient temperature, relative humidity, and daylength was 30.04 ± 2.05 °C, 78.39 ± 3.01%, and 13.20 ± 0.73 h, respectively) and hygiene conditions. Each doe was kept in an individual standard galvanized battery cage (60 cm L × 55 cm W × 40 cm H) equipped with feeders and automatic drinkers. Rabbit does were fed a pellet diet (18.32% crude protein and 10.76 MJ/kg digestible energy) containing 18% barley, 25% wheat bran, 6% yellow corn, 18% soybean, 27% alfalfa hay, 3% molasses, 1% di-calcium phosphate, and 2% NaCl and premix, meeting their daily nutritional requirements as recommended by the National Research Council [26]. Rabbit does were randomly divided into four experimental groups (*n* = 26 per group): 0 mg/kg body weight (BW) free ME ©, 50 mg/kg BW free ME (FME), 25 mg/kg BW nano-encapsulated ME (HNME), and 10 mg/kg BW nano-encapsulated ME (LNME). The treatments were orally administered to rabbit does daily for a complete reproductive cycle (around 75 days), including premating (10 days pre-insemination), mating (insemination day), pregnancy (30 days), and lactation (30 days). The dose of ME for each rabbit doe was individually added to 50 mL of water, which is less than the daily consumption rate to ensure full consumption of each dose. Then clean tap water was offered to each doe *ad libitum*. This process was daily repeated during the entire experimental period. Estrus synchronization was accomplished by administering 25 IU of equine chorionic gonadotropin (Gonaser^®^, Hipra, Spain) via intramuscular injection (IM) to each doe, which was followed 48 h later by the IM administration of 0.8 μg of gonadotropin-releasing hormone (0.8 μg buserelin; Receptal, Boxmeer, Holland) to induce ovulation. Does were immediately artificially inseminated with 0.2 mL (15 × 10^6^ sperm/insemination) of fresh diluted (1:5) pooled semen collected from previously proven-fertile rabbit bucks [27].

### 2.4. Physiological Variables

Each rabbit doe was weighed weekly in the morning before offering feed. The feed intake (g/day) was calculated daily by subtracting the unconsumed feed from the total amount of offered feed [28].

### 2.5. Blood Sampling and Analysis

Blood samples were collected from the marginal ear veins of eight randomly selected does using heparinized tubes (blood collection vacuum tubes, REF: G40111, NEW VAC, China) during premating (10 days after the beginning of the treatments), mating (insemination day), pregnancy (days 10 and 20 of pregnancy), and lactation (day 7). Each sample was divided into whole blood and separated blood plasma (2000× *g* for 20 min at −4 °C to obtain plasma) for immune variable and biochemical attribute analyses.

#### 2.5.1. Blood Biochemical Attributes

Blood plasma glucose concentrations were colorimetrically measured using commercial test kits (SPINREACT, Girona, Spain). Blood plasma β-HB and NEFA concentrations were determined via the kinetic enzymatic method using commercial kits (DiaSys Diagnostic Systems, Holzheim, Germany).

#### 2.5.2. Immune Variables

Whole blood samples were used to assess immune variables, namely white blood cells (WBCs), WBC differential count, and phagocytic activity. To determine the phagocytic activity, a mixture (1:1) of whole blood and *Staphylococcus* *albus* (1.0 × 10^5^ cells/mL) in phosphate-buffered solution (pH = 7.2) was incubated for 30 min at 37 °C. A smear of the mixture was prepared, dried, and fixed with methanol for 30 min. The smear was processed via Levowitz–Weber staining for 2 min and washed three times with distilled water. Phagocytic cells with engulfed bacteria were counted using a light microscope at 100 × magnification, and phagocytic activity was calculated as the percentage of phagocytic cells containing bacterial cells. The plasma lysozyme activity was determined by mixing a 50-μL plasma sample with 3 mL of *Micrococcus lysodekticus* bacterial suspension. The absorbance of the mixture was measured at 570 nm directly after plasma addition (A_1_) and again after incubation for 30 min (A_2_) at 37 °C. The plasma lysozyme activity was calculated using the following formula: lysozyme activity = (A_1_–A_2_)/A_2_ [29].

Interleukin-1β (IL-1β) in the blood plasma samples was determined using a commercial kit (Cat. No. MBS262525, MyBioSource, Inc., San Diego, CA, USA). The sensitivity of the method was 5 pg/mL, and the intra- and inter-assay precisions were ≥ 8% and ≥12%, respectively. Immunoglobulin G (IgG) and immunoglobulin M (IgM) were assessed by the colorimetric method using commercial kits (IBL America Immuno-Biological Laboratories, Inc., Spring Lake Park MN, USA); the sensitivity and specificity of the assays exceeded 96%.

### 2.6. Colostrum and Milk Analysis

#### 2.6.1. Colostrum Collection and Analysis

Colostrum samples were collected from eight randomly selected does within 8 h post-parturition. For this purpose, nests were checked three times a day, and colostrum samples were collected once the parturition process was completed. Colostrum samples were collected manually by gently massaging the mammary gland of the doe. The concentrations of colostrum immunoglobulin (Ig) fractions (IgM, IgA, IgG, IgE, and IgD) were determined using commercial kits (IBL America Immuno-Biological Laboratories, Inc., Spring Lake Park, MN, USA).

#### 2.6.2. Milk Collection and Analysis

Milk samples were collected on days 7, 14, and 21 using an ‘air vacuum pump’ from all nipples of the mammary gland and milk yield was calculated by the weight-suckle-weight method [1]. Briefly, kits were separated from their dams for 24 h to prevent free suckling. Then, does were treated with oxytocin to stimulate milk ejection, and a 10 mL sample was obtained. Next, does were allowed to nurse their kids. The kits were weighed before suckling and again after suckling. The sum of the difference between the weight of kits before and after suckling and the weight of the collected milk samples represents the milk yield of each doe.

Milk samples were diluted with deionized distilled water at a proportion of 1:2 to facilitate the analysis. Approximately 10 g of the diluted sample was weighed into a silica crucible and placed in an oven at 70 °C until dry. The oven temperature was then increased to 105 °C for 3 h until a constant weight was reached to obtain the total solids. The total moisture was calculated as the difference between the fresh sample weight and the total solids weight. The contents of protein, fat, and total solids in milk samples were determined according to [30] and the energy concentration in milk was estimated [31]. Milk FAs were extracted and identified as described in Section 2.2.

### 2.7. Productive and Reproductive Performance

Fertility and pregnancy output variables including conception rate, parturition rate, and litter size and litter weight of kits at birth (day 0 of the age of kits), and at weaning (day 30 of the age of kits) were recorded. The conception rate ([number of does diagnosed positive on day 10/number of inseminated does] × 100), parturition rate ([number of delivered does/number of inseminated does] × 100), litter size at birth and weaning (total rabbits born and weaned per each doe), and litter weight at birth and weaning were recorded [27].

### 2.8. Statistical Analysis

The Statistical Analysis Software package (Version 8. Cary, NC, USA; 2001) was used to analyze all results. Variables assessed more than once (i.e., the reproductive cycle stages) were analyzed using the MIXED procedure for repeated measurement with a model considering fixed and random effects. The fixed effects of treatment (C, FME, HNME, and LNME), status (premating, mating, pregnancy, and lactation), and the treatment/status interaction on physiological, immunological, and biochemical variables were assessed. The rabbit does effect was introduced as a random factor. One-way ANOVA was used to assess the treatment effects on colostrum, milk, and litter characteristics (number and weight). The chi-square test was used to assess the effects of treatments on conception and parturition rates. Duncan’s multiple range test was used to detect differences among treatment means. Results are presented as the least square mean (± pooled standard error of the mean [SEM]). The significance level of the statistical analysis tests was set at *p* < 0.05.

## 3. Results

### 3.1. Fatty Acid Profile of ME and EE

The ME FA profile analysis identified 30 FAs (Table 1). The major detected FAs were palmitic acid methyl ester C16:0, oleic acid methyl ester C18:1n-9, lignoceric acid methyl ester C24:0, gamma-linolenic acid methyl ester C18:3n-6, caprylic acid methyl ester C8:0, behenic acid methyl ester C22:0, and docosahexaenoic acid methyl ester C22:6n-3. The concentration of total UFAs was higher than the total SFAs. Odd-chain FAs were also detected (Table 1).

The EE of alginate–CaCl_2_ for Me FAs reached 100% for 14 FAs and ranged from 0 to 57% for the remaining FAs. The range of EE of alginate–CaCl_2_ for Me FAs was between 49.1% to 80.45% (Table 2).

### 3.2. BW, Feed Intake, and Energy-Related Metabolites

**Figure 1:**
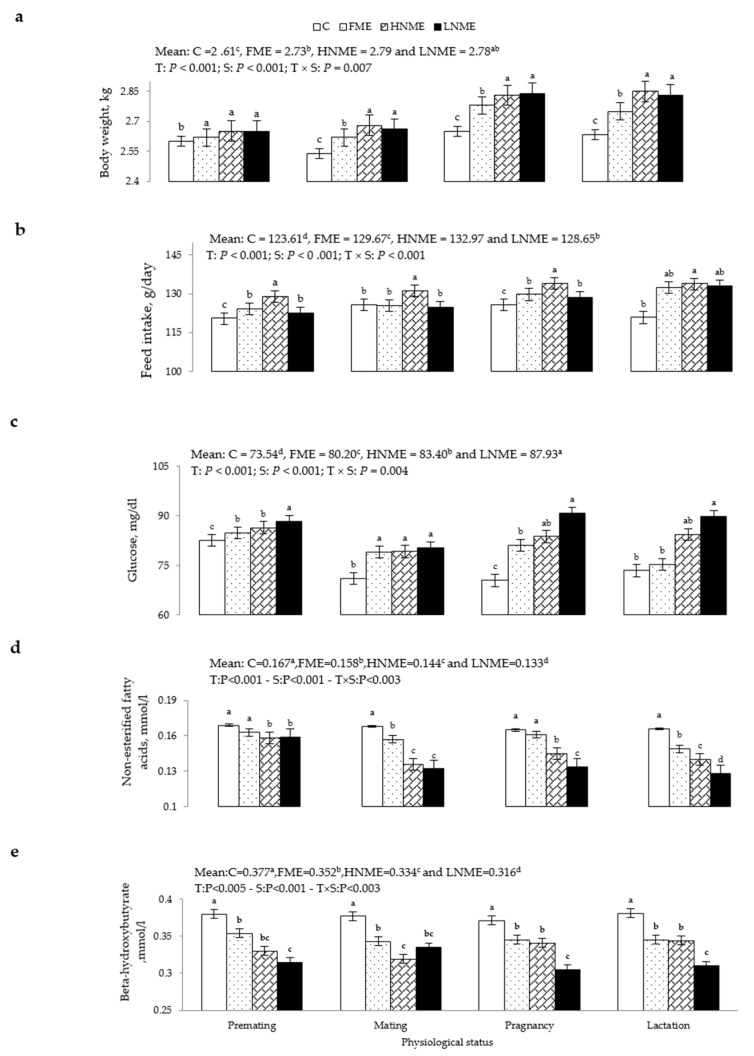
Means (± SEM) treatment by physiological status effects on body weight (**a**), feed intake (**b**), blood plasma glucose (**c**), non-esterified free fatty acids (**d**), and β-hydroxybutyra© (**e**) of multiparous rabbit does supplemented with 0 mg/kg BW (C), 50 mg/kg BW free ME (FME), 25 mg/kg BW nano-encapsulated ME (HNME), and 10 mg/kg BW nano-encapsulated ME (LNME). The means of different treatments within the same physiological status with different lowercase letter superscripts differ significantly (*p* < 0.05).

The effects of different ME treatments on the BW, feed intake, blood plasma glucose, β-HB, and NEFAs of rabbit does during the experimental period are shown in Figure 1. Compared with the C group, all ME treatments significantly increased BW (*p* < 0.001, Figure 1a) and feed intake (*p* < 0.001, Figure 1b), and the highest values were observed in the HNME treatment. The ME treatments significantly increased blood plasma glucose concentrations (*p* < 0.001, Figure 1c) compared with the C group, and the highest values were observed in the LNME and HNME treatments. This effect began at mating and continued through pregnancy and lactation. The ME treatments significantly decreased the concentrations of blood plasma NEFAs (*p* < 0.001, Figure 1d) and β-HB (*p* < 0.005, Figure 1e) compared with the C group, and the lowest values were observed in the LNME treatment. These effects started at mating and continued during pregnancy and lactation periods, except the effect on NEFAs, which started earlier at premating.

### 3.3. Blood Immune Variables

**Figure 2:**
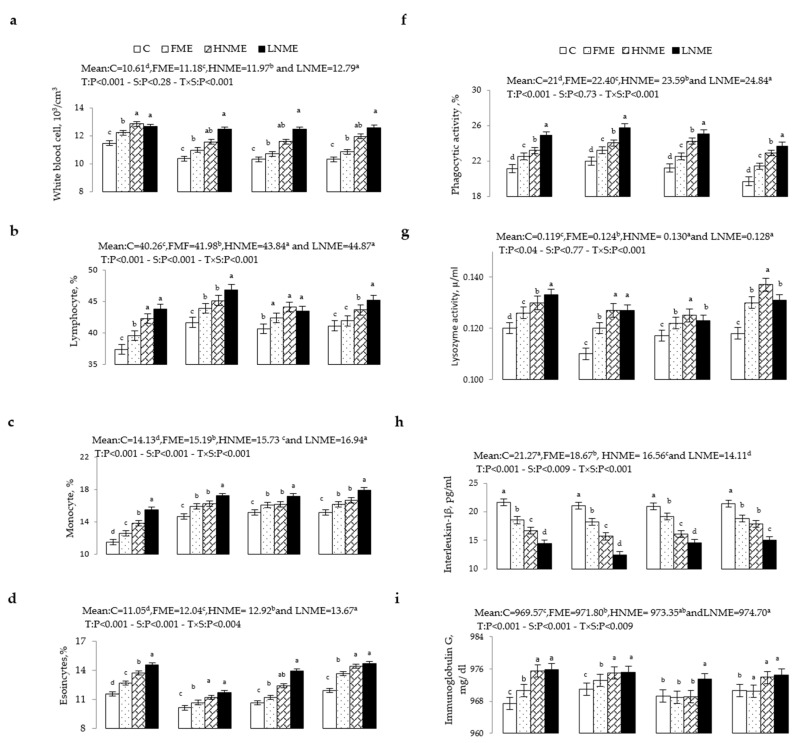

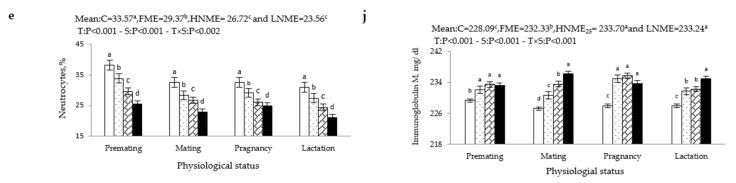
Means (± SEM) treatment by physiological status interaction effects on immunity variables; white blood cell (**a**), lymphocytes (**b**), monocytes (**c**), eosinocytes (**d**), neutro©es (**e**), phagocytic activity (**f**), lysozyme activity (**g**), interleukin-1β (**h**), immunoglobulin G (**i**) and immunoglobulin M (**j**) of multiparous rabbit does supplemented with 0 mg/kg BW (C), 50 mg/kg BW free ME (FME), 25 mg/kg BW nano-encapsulated ME (HNME), and 10 mg/kg BW nano-encapsulated ME (LNME). The means of different treatments within the same physiological status with different lowercase letter superscripts differ significantly (*p* < 0.05).

The effects of ME treatments on the blood immune system variables of rabbit does during the experimental period are shown in Figure 2a–e. Compared with the C group, all ME treatments increased numbers (*p* < 0.001) of WBCs and percentages (*p* < 0.001) of lymphocytes, monocytes, eosinophils, phagocytic activity, and lysozyme activity (*p* < 0.04), whereas the percentage of neutrophils was decreased (*p* < 0.001). The largest changes were detected in HNME and LNME, followed by FME. These changes were observed at different physiological stages, starting at premating. Compared with the C group, the ME treatments decreased (*p* < 0.001) blood plasma interleukin concentrations at all physiological stages and the lowest value was observed in the LNME treatment. The ME treatments significantly increased (*p* < 0.001) blood plasma immunoglobulin G and M compared with the C group; LNME treatments resulted in the highest value. These effects started at mating and continued through pregnancy and lactation.

### 3.4. Colostrum Immunoglobulin, Milk Yield, and Milk Composition

The effects of ME treatments on colostrum immunoglobulin of rabbit does during the experimental period are shown in Table 3. Compared with the C group, the ME treatments significantly increased (*p* = 0.001) the concentrations of colostrum immunoglobulin (IgG, IgA, IgM, and IgD) but did not affect (*p* = 0.160) IgE concentrations. The highest values were observed in LNME and HNME treatments, followed by FME. The ME treatments significantly increased milk yield (*p* = 0.001) and milk composition (protein, *p* = 0.001; total solids, *p* = 0.001; and energy milk contents, *p* = 0.005) and tended to increase fat content (*p* = 0.07) compared to the C group. The highest values were observed in the LNME treatment.

### 3.5. Milk Fatty Acid Profile and Fatty Acid Health Indices

From the 20 FAs studied, 13 were affected by the nano-encapsulated ME treatments (Table 4). For SFAs, all ME treatments significantly increased the concentrations of C16:0 to C20:0 and significantly decreased C15:0 compared with the C group. However, the ME-treatments significantly decreased the concentration of C15:0 in milk compared with the C group. All ME treatments significantly increased milk USFAs (*p* = 0.007), MUFAs (C16:1n-7 and C18:1n-9; *p* = 0.001), PUFAs, omega-6 (C18:2n-6, C20:4n-6; *p* = 0.014) compared with the C group. Higher concentrations of omega-3 FAs (C18:2n-3, C18:3n-3, C20:5n-3, and C22:6n-3) were recorded in the LNME and HNME treatments compared with the FME and C treatments (Table 4).

### 3.6. Reproductive Performance

The effects of ME treatments on the reproductive performance of rabbit does during the experimental period are shown in Table 5. Compared with the C group, the ME treatments significantly increased conception (*p* = 0.004) and parturition (*p* = 0.003) rates, the total litter sizes at birth and weaning (*p* = 0.003 and *p* < 0.001, respectively), and the litter weights at birth and weaning (*p* = 0.005 and *p* < 0.001, respectively); the highest values were observed in LNME and HNME treatments, followed by the FME treatment (Table 5).

## 4. Discussion

Recent research has focused on the development of phytogenic-based feed additives due to their impressive biological activities and safety for humans, animals, and the environment. However, the effectiveness of such feed additives is challenged by the sensitivity of these compounds to different environmental and industrial factors, causing reductions in nutritional value, bioavailability, product quality, and stability. In this context, encapsulation technology presents an innovative solution for maintaining the biological activities of sensitive active components [21,32].

In this study, ME was selected as a source of FAs because several studies have reported the richness of ME with biologically active FAs, specifically PUFAs [19,33]. PUFAs contain many double bonds, which make them highly susceptible to lipid oxidation, which diminishes their nutritional value and biological effects. Encapsulation can protect high n3/n6-containing FA products from oxidation reactions upon exposure to oxidation-inducing factors, such as oxygen, metal ions, high temperatures, and light [21]. This effect was confirmed in this study. Alginate proved to be an excellent encapsulating material that encapsulated 71.3% of SFAs, 69.62% of UFAs, 77.64% of MUFAs, and 60.26% of PUFAs in ME. Among the biopolymer materials used for encapsulation processes, alginate has the potential to form a film or coating due to its colloidal properties. Alginate has been found to effectively coat materials with different active components, including lipid sources, such as essential oil, fish oil, and plant oil (seed, leaves, flower) [32,34,35]. In this study, although all ME treatments improved the metabolism and productive and reproductive traits of rabbit does, the encapsulated ME resulted in better responses and productivity (milk production and reproductive performance), even at the lower ME concentration. These findings support the benefits of encapsulation for maintaining the biological activities of natural phytogenic compounds intended for use as feed additives. Similar results were previously obtained by [22,36].

In this study, we compared the effects of ME in free and encapsulated forms on the metabolic status and immunity of rabbit does during a whole reproductive cycle (from premating to weaning). We hypothesized that ME FAs might change colostrum/milk yield and composition to improve the viability and growth performance of litters. Rabbits are typically bred in intensive production systems, meaning they are pregnant, suckling, or both for most of their lifetime. These reproductive events are very costly in terms of energy [8].

In this study, we observed positive effects of ME supplementation on BW and metabolism. These effects may be mediated by different mechanisms. The first mechanism may be related to improved BW and metabolism due to changes in the feed intake of rabbit does, specifically during pregnancy and lactation. In this study, rabbit does supplemented with ME showed higher feed intake during pregnancy and lactation periods. Normally, doe feed intake decreases at the end of pregnancy due to the limited space available in the gastrointestinal tract and the lack of available carbohydrates [16], resulting in a negative energy balance due to the transfer of the body fat mass into the fetuses. During lactation, the feed intake of females increases very rapidly after kindling (60–75%), but this increase is insufficient to cover the requirements due to maintenance and milk production. Thus, a very negative energy balance and considerable mobilization of body fat are often observed during the first lactation. About 80% of the energy for reproduction comes from feed intake and about 20% from fat mobilization. Moreover, the energy deficit increases when females are concurrently pregnant and lactating [1]. The improved feed intake in ME-supplemented groups can be attributed to the effect of some FAs on energy-regulating hormones, such as leptin. Leptin suppresses food intake, inducing weight loss [15,16]. The inclusion of EPA and DHA in pregnant and lactating rabbit females is associated with decreased leptin levels (higher leptinemia) [37], which may partially explain the increased feed intake of ME-supplemented rabbit does. The increased feed intake could also be related to improved feed digestibility and increased flow rate of feed bolus as reported previously by [38], allowing does to consume more feed.

The second mechanism related to the improved metabolism of ME-treated does may be the increased availability of energy-yielding metabolites. All ME-supplemented rabbit does had better energy status, as indicated by higher body weights and blood plasma glucose and lower lipid metabolites (NEFAs and β-HB) during reproductive events, specifically pregnancy and lactation. In rabbits, NEFAs, glucose, and long-chain FAs play a role in the relationship between energy balance and reproductive efficiency [39]. During the post-partum period, mammary glands and fetoplacental units use substrates, such as glucose, long-chain FAs, and free FAs as energy sources [2]. Thus, the lack of these substrates drives does to use body fat to meet the increased energy requirements of growing fetuses and/or milk lactation. In this study, ME supplementation improved the concentration of blood plasma glucose and provided FAs as energy sources. These findings are consistent with many previous studies reporting that ME is a good source of energy-yielding nutrients that can be easily used by animals for biological events, such as growth [40,41] and reproduction [22,33]. The increase in glucose concentrations in ME-treated rabbit does might be due to improved gluconeogenesis. Interestingly, glucose cannot be synthesized from FAs because they are cleaved by *β*-oxidation into acetyl coenzyme A (CoA), which subsequently enters the citric acid cycle and is oxidized to CO_2_. However, the last three carbon atoms of odd-chain FAs generate propionyl CoA during *β*-oxidation and are thus partly gluconeogenic. Because ME contains 13.43% of odd-FAs, this pathway provides a conceivable reason for increased glucose concentrations in this study [42].

In this study, ME supplementation not only improved the energy status of rabbit does but also improved their immune status. ME-supplemented rabbit does exhibited better innate (phagocytes and their phagocytic activity and lysozyme activity) and humor (immunoglobulin G & M) immunity indicators than non-supplemented rabbit does. These findings are in accordance with those reported in several previous studies [38,43,44]. In context, Isitua and Ibeh [45] reported that rabbits fed *Moringa* leaves showed a significant increase in CD4 (T-helper) cells, which evoke cell-mediated immunity and help B-cells to produce antibodies. The positive role of ME on immune system function can be ascribed to the presence of considerable quantities of various FAs in ME. Immune cells contain FAs and, thus, their reactivity and functioning can be modulated by the profile of dietary fats. Supplementary dietary FAs may influence the immune status via several mechanisms, such as the inhibition of the arachidonic acid (AA) metabolic process, production of anti-inflammatory mediators, modification of intracellular lipids, and activation of nuclear receptors. ME contains high concentrations of omega-3 FAs (11.38%), EPA, and DHA, which stimulate phagocytic activity by macrophages in addition to their anti-inflammatory, anti-proliferative and anti-atherosclerotic activities [10]. In this study, ME contained high concentrations of omega-6 FAs (13.55%), which are linked to inflammation, mainly because AA is the precursor of pro-inflammatory lipid mediators. However, the concentrations of IL-1B (an inflammatory factor) were lower in ME-treated rabbit does. This finding can be explained by the fact that ME naturally has the recommended ratio between omega-6 and omega-3 PUFAs, which should be from 3:1 to 1:1 to provide positive effects on immunity and health [46].

The positive energy and immune status of ME-supplemented rabbit does were reflected in their productive and reproductive performance. ME-supplemented rabbit does had better colostrum and milk yield and composition as well as enhanced fertility traits. Colostrum is a special type of milk formed during the last days of pregnancy and the first few days after birth. The main components of colostrum in rabbits are proteins and fats. Thus, the nutritional requirements for these components are elevated, specifically toward the end of pregnancy and the early stages of lactation. For example, the coefficients of variation in total protein concentration reach their maximum during the early stages of lactation (5–26%), and immunoglobulins account for most of the total protein in colostrum [47]. As reported in previous studies [38,47], we found that ME improved colostrum protein and fat contents due to its high amino acid and FA concentrations. The improved protein content and different immunoglobulin (IgG, IgA, and IgM) in colostrum reflects an increased availability of amino acids for immunoglobulin synthesis [48] and improves the passive transfer of immunoglobulin to the mammary gland. Moreover, some FAs identified in ME have been shown to improve immunoglobulin contents in colostrum; for example, colostrum IgG concentrations were increased in animals fed an n-3 PUFA-rich diet [9].

Supplementation of ME also improved both milk yield and milk composition, specifically protein and fat content. Given that the ME-treated rabbit does had increased BWs and blood metabolites (higher glucose and lower fat metabolites) than the control rabbit does during the energy-consuming lactation period, it can be said that ME played a role in supporting energy requirements and modulating metabolism in favor of milk production without negative effects on energy balance status. In fact, feeding either Moringa leaf or ME has been shown to increase milk production in many farm animals, including rabbits [49,50]. Moringa contains plenty of nutrients, such as amino acids, FAs, and vitamins, which are precursors for milk synthesis [51]. Moreover, it contains phytosterols, phytoestrogens that can boost both lactogenesis and mammogenesis [18]. Moringa is rich in phytosterols, such as sitosterol, stigmasterol, and campesterol, which are precursors for hormones. These phytochemicals, along with high amino acid contents, can enhance estrogen excretion, stimulating mammogenesis and milk production [20].

In the present study, all ME treatments significantly increased the concentrations of milk USFAs, MUFAs (C16:1n-7 and C18:1n-9), omega-6 PUFAs (conjugated linoleic acid [CLA] and AA), omega-3 PUFAs (linoleic acid [LA], alpha-linolenic acid, EPA, and DHA) compared to the C group. As identified from the ME FA profile, ME has considerable concentrations of omega-3 PUFAs (EPA and DHA) and omega-6 PUFAs (LA, gamma-linolenic acid, and dihomo-gamma-linolenic acid), which are implicated in PUFA long-chain biosynthetic pathways. LA is the main precursor for omega-6 FA pathways; it can be used as a precursor for longer omega-6 PUFAs via the effects of elongase and desaturase enzymes [46]. This pathway seems to be enhanced in this study, as ME-treated rabbit does had higher concentrations of longer omega-6 PUFAs (CLA and AA) in their milk. The same trend was observed for omega-3 PUFAs, and omega-3 EPA can be used as a precursor for omega-3 DHA biosynthesis [16,17]. Overall, ME supplementation to rabbit does during lactation improved FA bioavailability to the mammary gland and activated FA biosynthetic pathways, leading to different FA profiles in the milk of ME-supplemented rabbit does. These positive effects might also be related to the improved energy status and the availability of energy-yielding nutrients, such as glucose, during lactation. It has been confirmed that the FA elongation process can be stopped if glucose, the acetyl-CoA source, is not adequately available [2,4].

Results of the present study confirmed the superior reproductive performance of the ME-supplemented rabbit does, which had better fertility and pregnancy outcomes than the non-supplemented (C) rabbit does. The positive effect of ME on the energy status of rabbit does is needed to achieve better reproductive performance. Several studies reported that higher BWs of rabbit does around mating time and early pregnancy are associated with better artificial insemination outputs [52,53]. In addition to the positive role of ME on body weight and metabolism, the unique FA composition of ME might have other positive effects. As reported in other studies, FA-rich diets can improve fertility and pregnancy outcomes in rabbits [15,46]. In fact, FAs are an important element for many reproductive events through various modes of action. Numerous FAs can positively influence reproduction by altering the ovarian follicles and corpus luteum function via improved energy status and increasing precursor levels for the synthesis of reproductive steroids and prostaglandins [54]. They also improve the competence of oocytes and boost embryo development [55] and pregnancy and fetal development [56]. For example, PUFAs, such as EPA and DHA can improve the quality of embryos by decreasing apoptosis rates and improving cell membrane integrity [15,57]. Moreover, omega-3 PUFAs are associated with low PGF2α production in uterine and placental tissues, decreasing the susceptibility to abortion and/or preterm parturition [58].

In this study, the ME-treated rabbit does had better pregnancy outcomes (litter sizes and weights at birth and weaning) than the control rabbit does. The developing fetuses and the offspring in the first days of lactation are totally dependent on their dams to provide the nutrition required for growth and development. Thus, such positive effects of ME on pregnancy outcomes might begin early and be related to improved oocyte quality, embryo development, and implantation during the early stages of pregnancy by providing specific FAs and/or energy sources. Additionally, during the later stages of pregnancy, most fetal lipids are delivered from maternal circulation through the placenta and are obtained from the diet or fat lipolysis [59], constituting the fat deposits on which the survival of the newborn depends. In this study, no decreases in dams body weight were observed in the ME-treated rabbit does. This finding leads us to speculate that ME was effective to support metabolism during pregnancy, providing the lipid/FA requirements for fetuses without depleting the dams’ fat stores. Because rabbit kits are altricial, the improved pregnancy outcomes may also be related to the improved growth rate and viability of litters after birth [18]. ME improved both colostrum immunoglobulin contents and milk production and composition and increased concentrations of some FAs that are important for boosting litter growth and health. Focusing on the important FAs for rabbit kit growth and development, higher EPA and DHA contents in milk were associated with the development of the nervous system because these FAs are used for membrane phospholipid synthesis [15,18]. Other FAs, such as AA, have been shown to boost the immune system and health status of kits [14].

## 5. Conclusions

In the present study, ME FAs served as a good source of energy and provided functional FAs for the ideal completion of specific physiological events related to milk production and reproduction. These effects were reflected in the improved metabolism, immune status, milk production, and reproductive performance of rabbit does at different reproductive stages. These effects were observed in rabbits supplemented with free and encapsulated forms of ME. However, the nano-encapsulated form allowed for an 80% reduction (10 mg/kg BW) in the optimal dose (50 mg/kg BW) without affecting the treatment efficiency, highlighting the importance of nano-encapsulation for improving FA bioavailability.

## Figures and Tables

**Table 1:** Fatty acids (FAs) profile and encapsulation efficiency of each individual identified FA of nano-encapsulated *Moringa* leaves ethanolic extract (ME).

FAs	FA, g/100 g FA Methyl Esters
Caprylic acid methyl ester, C8:0	5.30
Capric acid methyl ester, C10:0	0.61
Undecanoic acid methyl ester, C11:0	1.01
Lauric acid methyl ester, C12:0	0.63
Tridecanoic acid methyl ester, C13:0	0.64
Myristic acid methyl ester, C14:0	1.18
Pentadecanoic acid methyl ester, C15:0	0.81
Palmitic acid methyl ester, C16:0	11.45
Heptadecanoic acid methyl ester, C17:0	1.45
Stearic acid methyl ester, C18:0	3.72
Arachidic acid methyl ester, C20:0	1.97
Heneicosanoic acid methyl ester, C21:0	2.55
Behenoic acid methyl ester, C22:0	5.14
Tricosanoic acid methyl ester, C23:0	3.26
Lignoceric acid methyl ester, C24:0	6.02
Myristoleic acid methyl ester, C14:1n-9	1.53
Pentadecenoic acid methyl ester, C15:1n-5	1.71
Palmitoleic acid methyl ester, C16:1n-7	2.12
Heptadecenoic acid methyl ester, C17:1n-7	2.00
Oleic acid methyl ester, C18:1n-9	9.04
Elaidic acid methyl ester, C18:1n-9t	3.82
Eicosenoic acid methyl ester, C20:1n-9	2.62
Erucic acid methyl ester, C22:1n-9	2.57
Nervonic acid methyl ester, C24:1n-9	3.93
Linolenic acid methyl ester (LA), C18:2n-6	3.68
Gama-Linolenic acid methyl ester (GLA), C18:3n-6	5.98
Dihomo-gamma-linolenic acid (DGLA), C20:4n-6	3.89
Eicosatrienoic acid methyl ester (ETE), C20:3n-3	3.53
Eicosapentaenoic acid methyl ester (EPA), C20:5n-3	3.40
Docosahexaenoic acid methyl ester (DHA), C22:6n-3	4.45
Saturated fatty acid	45.73
Unsaturated fatty acid	54.27
Mono-unsaturated fatty acid	29.34
Poly-unsaturated fatty acid	24.93
Poly-unsaturated fatty acid/Saturated fatty acid	0.54
Total odd FAs	13.43
Omega-3 FAs	11.38
Omega-6 FAs	13.55
Omega-9 FAs	21.98
Omega-6 FAs/Omega-3 FAs	1.19

**Table 2:** Encapsulation efficiency of alginate-CaCL_2_ for moringa extract fatty acids (FAs).

FAs Category	Encapsulation Efficiency ^1^, %
Saturated FAs	71.03
Unsaturated FAs	69.62
Mono-unsaturated FAs	77.64
Poly-unsaturated FAs	60.26
Total odd FAs	80.45
Omega-3 FAs	71.4
Omega-6 FAs	49.1
Omega-9 FAs	68.70

^1^ Encapsulation efficiency (EE, %) = FA concentration in raw MLEE-FA concentration in MLEE supernatant/ FA concentration in raw MLEE × 100.

**Table 3:** Effects of free and nano-encapsulated *Moringa oleifera* leaf ethanolic extract (ME) supplementations on colostrum, milk yield and milk composition of multiparous rabbit does during the experimental period (mean ± SEM).

Variable	Treatment ^1^				SEM	*p* Value
	C	FME	HNME	LNME		
Colostrum immunoglobulin, mg/dL						
Immunoglobulin M	225.32 ^c^	230.85 ^b^	237.15 ^a^	232.14 ^b^	2.27	0.001
Immunoglobulin A	73.22 ^b^	75.23 ^a^	76.89 ^a^	75.56 ^a^	0.91	0.001
Immunoglobulin G	964.20 ^c^	968.21 ^b^	972.54 ^a^	968.23 ^b^	2.35	0.001
Immunoglobulin E	12.99	13.22	13.52	13.39	0.07	0.160
Immunoglobulin D	28.86 ^c^	31.83 ^b^	35.52 ^a^	35.53 ^a^	0.82	0.001
Milk yield and composition						
Milk yield, g/day	117.50 ^c^	159.44 ^b^	161.52 ^ab^	169.86 ^a^	2.46	0.001
Milk Composition, %						
Protein	11.79 ^d^	12.33 ^c^	12.68 ^b^	13.39 ^a^	0.02	0.001
Fat	13.38	13.50	14.12	14.69	0.33	0.07
Total solids	27.91 ^c^	29.54 ^bc^	31.77 ^ab^	32.51 ^a^	2.23	0.001
Energy, MJ/kg	8.50 ^c^	8.56 ^bc^	9.04 ^ab^	9.41 ^a^	0.06	0.005

^1^ Multiparous rabbit supplemented with 0 kg BW (C), 50 mg/kg BW free ME (FME), 25 mg/kg BW nano-encapsulated ME (HNME), and 10 mg/kg BW nano-encapsulated ME (LNME). Means within the raw having different superscripts (a, b, c) differ significantly (*p* < 0.05).

**Table 4:** Effects of free and nano-encapsulated *Moringa oleifera* leaf ethanolic extract (ME) supplementations on milk fatty acids (FAs) profile of multiparous rabbit does during the experimental period (mean ± SEM).

FAs, g/100 g FA Methyl Esters	Treatment ^1^				SEM	*p*-Value
	C	FME	HNME	LNME		
Butyric acid, C4:0	0.101	0.112	0.110	0.108	0.002	0.616
Caproic acid methyl ester, C6:0	0.410	0.410	0.411	0.403	0.001	0.378
Caprylic acid methyl ester, C8:0	25.59	25.97	26.27	26.13	0.11	0.167
Capric acid methyl ester, C10:0	21.76	21.59	21.74	22.18	0.12	0.476
Lauric acid methyl ester, C12:0	2.66	2.67	2.77	2.79	0.08	0.938
Myristic acid methyl ester, C14:0	1.55	1.54	1.57	1.57	0.01	0.729
Pentadecanoic cid methyl ester, C15:0	0.827 ^a^	0.757 ^b^	0.816 ^a^	0.773 ^ab^	0.01	0.039
Palmitic acid methyl ester, C16:0	12.94 ^ab^	13.30 ^a^	12.56 ^b^	12.79 ^b^	0.01	0.030
Heptadecanoic acid methyl ester, C17:0	0.713 ^c^	0.755 ^b^	0.744 ^bc^	0.789 ^a^	0.009	0.006
Stearic acid methyl ester, C18:0	2.66 ^b^	3.00 ^a^	2.78 ^b^	2.80 ^b^	0.04	0.005
Arachidic acid methyl ester, C20:0	0.125 ^b^	0.182 ^ab^	0.179 ^ab^	0.221 ^a^	0.01	0.013
Myristoleic acid methyl ester, C14:1 n-9	0.125 ^b^	0.159 ^a^	0.164 ^a^	0.161 ^a^	0.01	0.021
Palmitoleic acid methyl ester, C16:1 n-7	1.52 ^c^	1.70 ^a^	1.66 ^ab^	1.63 ^b^	0.02	0.001
Oleic acid methyl ester, C18:1 n-9	11.39 ^b^	11.39 ^b^	11.42 ^ab^	11.43 ^a^	0.007	0.004
Conjugated Linoleic Acid (CLA), C18:2 n-3	0.074 ^c^	0.080 ^b^	0.085 ^a^	0.086 ^a^	0.002	0.001
Linolenic acid methyl ester (LA), C18:2 n-6	12.21 ^b^	12.49 ^a^	12.60 ^a^	12.56 ^a^	0.05	0.030
α-Linolenic acid methyl ester (ALA), C18:3 n-3	2.47 ^c^	2.50 ^b^	2.51 ^b^	2.55 ^a^	0.01	0.007
Arachidonic acid methyl ester(ARA), C20:4 n-6	0.537 ^ab^	0.523 ^b^	0.534 ^b^	0.548 ^a^	0.003	0.015
Eicosapentaenoic acid methyl ester(EPA), C20:5n-3	0.049 ^c^	0.068 ^b^	0.074 ^a^	0.073 ^a^	0.004	0.001
Docosahexaenoic acid(DHA), C22:6n-3	0.061 ^b^	0.065 ^b^	0.070 ^a^	0.076 ^a^	0.004	0.001
Degree of FAs saturation, g/100 g FA methyl ester						
Saturated FAs	72.27	71.72	71.62	71.70	0.22	0.282
Unsaturated FAs	27.73 ^b^	28.28 ^a^	28.38 ^a^	28.30 ^a^	0.08	0.007
Monounsaturated FAs	13.04 ^c^	13.28 ^a^	13.26 ^ab^	13.19 ^b^	0.03	0.001
Polyunsaturated FAs	14.69 ^b^	15.00 ^a^	15.12 ^a^	15.11 ^a^	0.06	0.014
Unsaturated FAs / Saturated FAs	0.400	0.402	0.405	0.401	0.001	0.552

^1^ Multiparous rabbit supplemented with 0 mg/kg BW (C), 50 mg/kg BW free ME (FME), 25 mg/kg BW nano-encapsulated ME (HNME), and 10 mg/kg BW nano-encapsulated ME (LNME). Means within the raw having different superscripts (a, b, c) differ significantly (*p* < 0.05).

**Table 5:** Effects of free and nano-encapsulated *Moringa oleifera* leaf ethanolic extract (ME) supplementations on the reproductive performance of multiparous rabbit does during the experimental period (mean ± SEM).

Variable	Treatment ^1^				SEM	*p*-Value
	C	FME	HNME	LNME		
Conception rate, %	76.9 ^c^ (20/26)	84.61 ^bc^ (22/26)	92.3 ^b^ (24/26)	96.15 ^a^ (25/26)	-	0.004
Parturition rate, %	69.23 ^c^ (18/26)	84.61 ^b^ (22/26)	88.46 ^ab^ (23/26)	92.3 ^a^ (24/26)	-	0.003
Litter size at birth	6.33 ^bc^	5.95 ^c^	7.17 ^b^	7.86 ^a^	2.4	0.004
No. live litter sizes	5.16 ^c^	5.90 ^ab^	6.65 ^b^	7.34 ^a^	3.7	0.003
No. dead litter sizes	1.17 ^a^	0.05 ^b^	0.52 ^ab^	0.52 ^ab^	2.2	0.130
Litter weight at birth, g	298.36 ^c^	324.17 ^b^	340.65 ^b^	409.30 ^a^	50.08	0.005
Litter size at weaning	5.27 ^c^	5.92 ^bc^	6.64 ^b^	7.21 ^a^	0.63	< 0.001
Litter weight at weaning, g	1699.1 ^c^	2376.2 ^b^	2796.8 ^ab^	3144.6 ^a^	174.46	< 0.001

^1^ Multiparous rabbit supplemented with 0 mg/kg BW (C), 50 mg/kg BW free ME (FME), 25 mg/kg BW nano-encapsulated ME (HNME), and 10 mg/kg BW nano-encapsulated ME (LNME). Means within the raw having different superscripts (a, b, c) differ significantly (*p* < 0.05).

## Data Availability

The data presented in this study are available on request from the corresponding author. The data are not publicly available because of privacy.
